# Same Involvement, Different Reasons: How Personality Factors and Organizations Contribute to Heavy Work Investment

**DOI:** 10.3390/ijerph17228550

**Published:** 2020-11-18

**Authors:** Greta Mazzetti, Dina Guglielmi, Wilmar B. Schaufeli

**Affiliations:** 1 Department of Educational Science, University of Bologna, Via Filippo Re, 6-40126 Bologna, Italy; dina.guglielmi@unibo.it; 2 Research Unit Occupational & Organizational Psychology and Professional Learning, KU Leuven, 3000 Leuven, Belgium; wilmar.schaufeli@kuleuven.be; 3 Department of Psychology, Utrecht University, 3584 CS Utrecht, The Netherlands

**Keywords:** workaholism, engagement, personality, organizational climate, job demands, overwork

## Abstract

The academic literature has drawn a clear distinction between a positive form (i.e., work engagement) and a negative form (i.e., workaholism) of heavy work investment (HWI). Nevertheless, the different weight of individual and situational factors contributing to their development was not thoroughly explored. This study aims to investigate the role of individual variables (i.e., obsessive–compulsive traits, achievement orientation, perfectionism, and conscientiousness) and situational factors (i.e., job demands and overwork climate) regarding engagement and workaholism simultaneously. Hypotheses were tested using a sample of 523 Italian employees. Results of structural equation modeling revealed that overwork climate and job demands were conversely related to engagement and workaholism, with job demand reporting the strongest association with workaholism. Furthermore, fear of failure was the only individual factor showing a significant and opposite relationship with workaholism and engagement. In contrast, perfectionism was positively associated with both forms of HWI. These results shed light on the potential effectiveness of intervention strategies focused on the employees and organizations in preventing workaholism and promoting engagement.

## 1. Introduction

As the tendency to devote a significant amount of time and effort to one’s work spreads across different organizations and occupational sectors, understanding the conditions driving heavy work investment (HWI) becomes crucial. To this end, several attempts have been made to disentangle healthy from harmful forms of HWI both from a theoretical and an empirical perspective [1]. This research strand focuses on exploring the similarities and differences between work engagement and workaholism, respectively, defined as positive and negative forms of HWI [2]. Both workaholics and engaged employees are highly focused and spend a great deal of time on their work tasks, but the motivation fueling this behavior and the outcomes stemming from it are strikingly different. Workaholism hinges on an overwhelming compulsion that translates into the combination of two components. The cognitive dimension entails an obsession with work (i.e., working compulsively), while the behavioral dimension describes the exceptional amount of time devoted to work beyond any economic or organizational purpose (i.e., working excessively).

Work engagement reflects a fulfilling work-related psychological state resulting from the combination of three key components. High levels of energy and resilience while working (i.e., vigor); feelings of enthusiasm, inspiration, and pride (i.e., dedication); and an intense concentration that involves finding it difficult to stop working (i.e., absorption) [3]. The latter dimension describes conduct shared by workaholics and engaged employees since they are both fully immersed in their work. Accordingly, empirical findings have consistently proved cross-loading of the absorption dimension on work engagement and workaholism [4].

Results of multi-trait multi-method studies have corroborated the assumption that engagement and workaholism are conceptually and empirically distinct forms of heavy involvement in one’s work [5]. A crucial point that determines the distinction between workaholism and engagement lies in the motivational dynamics involved. Workaholic employees are driven by a controlled motivation [6] that translates into compulsive work behavior intended to avoid others’ disfavor and comply with strict standards imposed by the internalization of social approval standards [7]. In contrast, engaged employees are prompted by an intrinsic motivation, which leads them to commit an extraordinary amount of time to their work not because they feel compelled to do so but because they feel gratified and satisfied by the job itself [8]. Owing to the different motivations behind these forms of HWI, the individual and organizational level outcomes are quite the opposite. Engaged employees experience high levels of satisfaction and exhibit extra-role behaviors, better team functioning, job performance, and lower rates of sickness-related absence and intention to quit their jobs [9]. Beyond the consequences in the organizational context, these employees report better social functioning, high life satisfaction, well-being, and general health [10]. Conversely, workaholism jeopardizes the quality of collaboration with colleagues and impairs employees’ performance through recurrent attempts to create additional demands that [11]. Mirroring the description of engagement outcomes, meta-analytic results suggest that individual results of workaholism include indicators of emotional exhaustion, mental distress, and health complaints [12].

Although the underlying motivational dynamics and opposite consequences would be sufficient to contend soundly the distinction between positive and negative types of HWI, a major research question relates to the divergence (or overlap) between their antecedents. Furthermore, each antecedent’s role could depict a different etiology, thus corroborating the distinctiveness between these constructs. Theoretical models in research on workaholism attribute great explanatory power to the trait theory approach [12]. According to this perspective, workaholism represents a dispositional behavioral pattern, and personality factors are a leading source of obsession with work [13]. This perspective suffers from an oversimplification of workaholism antecedents, assuming compulsive conduct toward work apart from environmental characteristics.

In contrast, the recent literature has emphasized the relevant roles of environmental factors in determining the onset of this negative form of HWI, especially in terms of an organizational climate fostering the behavioral component of workaholism [14]. In particular, Schaufeli [2] conducted a noteworthy attempt to explore the relative contributions of personality factors and work environment in explaining the development of both positive and negative forms of HWI by focusing on Big Five personality traits and overwork climate. The current study aims to take the research one step further by assessing the role of personality factors recognized as main individual antecedents of workaholism and situational factors related to HWI. In doing so, structural equation modeling allows observing each association independently from each other.

The prominent models of workaholism based on literature reviews and meta-analysis concur in identifying several personality factors as catalyzing features that mold workaholic employees: obsessive–compulsive traits, achievement orientation, perfectionism, and conscientiousness [12,15,16]. We also included work engagement, a positive form of strong investment toward one’s work, to assess the extent to which the same personality and situational factors play a role in both forms of HWI. To this purpose, personality and organizational factors were simultaneously evaluated to consider their associations with HWI as unique (i.e., controlled for the effect of the other set of variables).

## 2. Theoretical Background

### 2.1. Individual Antecedents of HWI

Workaholism is defined as an inner compulsion to allocate an excessive amount of time and energy to work because of an obsession with work-related activities [2]. This description suggests that workaholics are obsessed with their work or, in other words, are compulsive workers. Consistently, obsessive–compulsive traits have been identified as individual antecedents that predispose employees to become addicted to work [16]. Obsession involves intrusive and unwanted recurrent and persistent thoughts, while compulsion entails repetitive behaviors or mental acts that an individual rigidly performs to prevent or neutralize obsession and anxiety [17]. Accordingly, workaholic employees are driven by the obsessive need to achieve self-imposed extraordinary goals, stemming from internalizing external standards of self-worth and social approval [8]. Thus, their compulsive work behavior is intended to prevent the negative feelings of guilt and worthlessness they experience when they do not devote time to job tasks (i.e., avoiding motivation). In contrast, engaged employees are committed to pursuing relevant goals associated with personal satisfaction and a sense of personal effectiveness (i.e., approach motivation). Obsessive–compulsive traits, embracing the three facets of orderliness, rigidity, and superego, are related to the need to perform tasks in a precise way and with meticulous attention to detail on account of a great need for control [18]. Consequently, these traits can result in addictive behaviors enacted with the specific purpose of tackling or obviating this obsession. In line with the conceptualization of workaholism as a form of addiction, obsessive–compulsive traits can explain the recurrent investment of discretional time in work activities and the tendency to constantly think about work when engaged in other activities [12]. In terms of specific obsessive traits, orderliness refers to the tendency to be accurate, methodical, and well organized and to avoid procrastination [19]. Orderly and methodical individuals plan their activities with the utmost diligence and are likely to fail urgent tasks because they overly focus on unnecessary activities requiring long-term investment. Therefore, tendencies to engage in unsolicited work can be particularly pronounced when high commitment levels are prompted by a compulsive drive to work (i.e., workaholism). Rigidity entails the tendency to follow routines so tightly that individuals become unable to break out or revise their behavioral patterns. Employees characterized by high rigidity are stuck in their points of view, dislike change, and embrace any effort to alter or improve their habits only with extreme skepticism [20]. A further obsessive–compulsive trait is superego, defined as the personal belief of possessing an extraordinary sense of ethics and higher standards of right and wrong than others, thus perceiving oneself as responsible and trustworthy [21]. The assumption of a reverse association between obsessive–compulsive traits and the opposite forms of working hard relies on their different motivational foundations, described by Houlfort and colleagues [22]. Obsessive–compulsive traits are expected to positively relate to workaholism, defined by Vallerand and colleagues [23] as an “obsessive passion” for work stemming from the necessity to satisfy internalized external values, to feel important and valuable, and to prevent an undesirable sense of guilt. Conversely, it is reasonable to postulate a negative relationship between obsessive–compulsive traits and work engagement, a form of “harmonious passion” whereby employees intentionally decide to work due to the gratification they derive from it, without experiencing any compulsion [23]. Therefore, the following hypothesis was tested:

**Hypothesis** **1** **(H1).** *Obsessive–compulsive traits (orderliness, rigidity, and superego) are positively associated with workaholism and negatively related to work engagement.*

Achievement motivation is commonly defined as the individual urge to pursue and reach relevant goals [24]. More specifically, achievement motivation embraces two different motivational dynamics: hope of success and fear of failure [25]. According to this distinction, fear of failure entails acting under avoidance-achievement tendencies that fuel behaviors to minimize the chances of missing the target. Conversely, hope of success implies approach-oriented trends involve attempts to act proactively to maximize the chances of successfully meeting standards of excellence. In line with the motivational dynamic involved, fear of failure can lead to workaholism through overwork habits aimed at reducing feelings of anxiety and lessening negative emotions caused by the concern of missing the mark at work [26]. Workaholic employees may devote excessive energies and resources to avoid unpleasant feelings of discomfort and worthlessness whenever they are not working. Although the academic literature has linked the overarching construct of achievement motivation to workaholism, it is reasonable to assume that this relationship is particularly relevant for fear of failure dimension [27]. Workaholic employees are driven by a partial internalization process concerning external standards of social approval. These employees strengthen themselves with feelings of self-esteem and self-worth whenever they can fulfill these internalized standards. Accordingly, individuals who are motivated by a powerful fear of failure act to protect themselves against internalized cognitive schemas and beliefs regarding feelings of unworthiness, shame, and guilt that arise in response to failure [28]. In contrast, hope of success could explain the motivational dynamics underpinning work engagement.

Engaged employees are driven by a learning goal orientation, leading them to actively pursue work-related challenges, giving rise to opportunities to improve their skills [29]. Thus, they are inspired by the research of successful experiences that enable them to identify, develop, and employ existing resources in their jobs, thus fueling the favorable condition of high work engagement [30]. The hope of success dimension is characterized by a robust individual willingness to achieve significant results and the perception of oneself as a resourceful, capable person who can accomplish goals, can overcome potential obstacles and has the knowledge and skills necessary to identify strategies for implementing plans [31]. This explanation accounts for the motivational dynamic behind the personal experience of prominent levels of involvement and dedication in one’s work (i.e., work engagement), perceived as a means for achieving crucial learning goals. In line with these empirical findings and theoretical explanation, the current study hypothesized a different association between the two facets of achievement motivation (i.e., fear of failure and hope of success) and HWI (i.e., workaholism and work engagement). Hence, we developed the second hypothesis as follows:

**Hypothesis** **2** **(H2).** *Workaholism is positively related to the achievement motivation dimension labeled as fear of failure, while hope of success exhibits a significant association with work engagement.*

Perfectionism is defined as the combination of exceedingly excessive performance standards and intense apprehension based on extreme self-criticism [32]. Perfectionists cannot disrupt vicious cycles of self-harm, accompanied by disproportionate effort in tasks perceived as opportunities that can lead to complacency, disappointment, and failure [33]. Perfectionism plays a crucial role in workaholism development and translates into the inability to delegate tasks and the tendency to generate additional tasks, rather than complete them, for the purpose of persevering in investing time and effort in one’s work [4]. The literature on workaholism has traditionally highlighted the relationship of perfectionism with an obsession with work, to the extent that the renowned conceptualization of Spence and Robbins [34] considered perfectionism as a feature that distinguishes real workaholics from people who are enthusiastic about their work without experiencing a compulsive drive. The evidence of a close link between perfectionism and workaholism is reinforced by results suggesting that both perfectionistic strivings (i.e., self-oriented striving for perfection) and perfectionistic concerns (i.e., concerns over mistakes and external evaluations) are significant antecedents of workaholism [13]. Falco and colleagues [35] contributed to the current understanding of the role played by perfectionism by revealing that both self-oriented perfectionism (i.e., the inclination to set extremely high standards for oneself) and socially prescribed perfectionism (i.e., unrealistically high standards imposed by significant others) are significantly associated with workaholism. As perfectionist individuals are inclined to set inaccessible standards for themselves, they perceive no possibility for their performance to match expected standards. As a result, these employees tend to commit greater effort and time to their work, and it is reasonable to assume that this characteristic is conducive to greater workaholism. Research conducted on different occupational sectors (including the public sector, healthcare, education, law, and administration) suggests a positive association between engagement and perfectionism [36,37]. Although these studies were conducted across different professional roles and cultural contexts, they concurred in reporting a positive relationship between work engagement and the perfectionism component of perfectionistic strivings. This facet of perfectionism, also defined as personal standards perfectionism, fuels a self-oriented striving for perfection and the setting of almost unreachable personal criteria for one’s performance [38]. To comply with the standards of accuracy required for themselves, employees characterized by strong perfectionistic strivings tend to allocate an extraordinary amount of time to work tasks. Accordingly, empirical findings suggest that employees high in perfectionistic strivings attain notable task performance [39]. Overall, these employees are immersed in their jobs, perceived as a source of satisfaction stemming from well-defined accuracy requirements. Thus, it is reasonable to assume that perfectionism would show a strong association with higher work engagement levels. Based on the literature discussed, the following hypothesis was assessed:

**Hypothesis** **3** **(H3).** *Perfectionism is positively associated with both workaholism and work engagement. Thus, employees characterized by high perfectionism levels are expected to report higher scores on workaholism and engagement.*

Conscientiousness is one of the Big Five personality factors describing the combination of industriousness, perseverance, and a sense of duty [19]. Conscientious individuals are orderly, highly responsible, self-disciplined, and strongly committed to attaining their goals. Because conscientious individuals tend to be characterized by persistence and achievement orientation, this personality factor has been recognized as a main antecedent of workaholism [14,40]. Individuals reporting high conscientiousness believe that they can successfully affect their surrounding environment. They cultivate a compelling sense of duty and responsibility in their private and work lives and are committed to making significant efforts to pursue their goals. Accordingly, empirical findings show that three of the main characteristics of conscientiousness—self-discipline, reliability, and orderliness—are strongly related to workaholism [18]. The high perseverance that characterizes conscientious individuals has led to the assumption that this personality factor is also related to a positive form of HWI, i.e., work engagement. The intrinsic motivational make-up of engagement could easily explain this association. Indeed, conscientiousness is characterized by strong self-discipline that pushes individuals to complete their tasks diligently to accomplish the intended goal, rather than being motivated by any rewards potentially associated with successful performance [41]. With specific attention to the work domain, employees who exhibit prominent conscientiousness levels show a high focus on their work, combined with a remarkable readiness to fulfill their work duties. Thus, it is reasonable to assume that conscientiousness plays a prominent role concerning work behavior characterized by high investment, be it positive (i.e., work engagement) or harmful (i.e., workaholism). The following hypothesis was grounded in this reasoning and prior results:

**Hypothesis** **4** **(H4).** *Conscientiousness is positively related to both workaholism and work engagement. In other words, a greater level of conscientiousness is expected to relate to higher scores on both workaholism and engagement.*

### 2.2. Situational Antecedents of HWI

The construct of psychological climate depicts employees’ perceptions of their work environment (which reflect how they make sense of events occurring in the workplace), the expectations placed on their performance, the consequences associated with different behaviors, and consequently which behaviors are legitimated or even rewarded at work [42]. Based on the premise that the exceptional devotion of time to work can be encouraged by the perception of a climate sustaining overwork, Mazzetti, and colleagues [43] developed the Overwork Climate Scale (OWCS). This measure includes two features of the overwork climate. The first is the perception of a work environment characterized by management that expects employees to work beyond their formal working hours and during their leisure time, considering overtime work a requirement for career advancement (i.e., overwork endorsement). The second essential dimension of overwork climate is the view of overwork as a norm that does not need to be limited or compensated in the form of benefits and rewards. Previous studies provided evidence for a positive relationship between overwork climate components and workaholism, although they showed mixed results regarding the relationship between this type of climate and engagement. According to Mazzetti and colleagues [43], only the lack of rewards for overwork is negatively related to engagement. At the same time, Schaufeli [2] reported a non-significant association between engagement and both the climate dimensions of overwork endorsement and lacking overwork rewards. These results corroborate the assumption that organizations that ask employees to spend a massive portion of time working could encourage compulsive attitudes toward work (i.e., workaholism). From a theoretical perspective, a strong requirement to overinvest in one’s work represents an external constraint that drains employees’ energy, thus reducing their motivation to work, based on the pleasure they derive from it. This reasoning suggests the assumption of a negative relationship between overwork climate and engagement. According to this rationale, the following hypothesis was assessed:

**Hypothesis** **5** **(H5).** *Overwork climate is associated with higher levels of workaholism and lower levels of engagement. Workaholism is expected to be higher when employees perceive a greater endorsement of overwork and a lack of overwork rewards. In contrast, employees who perceive a climate that prompts them to overinvest in their job also experience lower engagement levels.*

Along with this first hypothesis, the perception of high job demands can stimulate excessive working, thus contributing to an increased risk of developing workaholism. Job demands are defined as psychological stressors consisting of requirements to perform mentally demanding and diverse tasks with a high work pace [44]. The need to tackle a heavy workload may provide an excuse for working extremely hard, thus encouraging workaholic behavior. Accordingly, previous results indicate that employees who perceive high work demands are at greater risk of developing workaholism than those experiencing a reasonable level of job demands [45]. The positive relationship between job demands and workaholism supports the definition of this negative form of HWI as an addiction that is considerably influenced by the environment (i.e., job demands) rather than a stable trait [46]. The current study hypothesized a negative association between job demands and work engagement. Employees faced with high job demands may suffer from continued draining of their energies and erosion of motivation, resulting in reduced engagement [47]. Thus, we formulated the following hypothesis:

**Hypothesis** **6** **(H6).** *Job demands are positively related to workaholism and negatively associated with engagement.*

## 3. Materials and Methods

### 3.1. Procedure and Participants

Participants in the current study were employees from an international company who filled in an online questionnaire as part of an occupational health survey. With the Human Resources division’s support, employees received an email with a link allowing them to fill in an online questionnaire. The first page of the questionnaire was a cover letter describing the overall purpose of the study. Participants’ anonymity and confidentiality were emphasized, as required by the Italian privacy law (Legislative Decree n. 101 of 10 August 2018), on personal data treatment guidelines. Furthermore, the current research followed the ethical standards for the research described in the latest version of the Declaration of Helsinki [48]. The letter also specified that participation was voluntary, and participants had the opportunity to withdraw from the study at any time without being required to justify their decision. A total of *n* = 523 employees returned the completed questionnaire (response rate: 78%). Most participants were women (72.5%), ranging in age from 19 to 63 years, with a mean age of 36.05 (*SD* = 8.65). Concerning the work role, most of the sample consisted of employees (64.5%), managers (22.7%), and supervisors (10.3%). Among the participants, 83% had a secondary school degree, 15% were graduates, and the remaining 2% had a higher qualification. Most of the sample had a permanent employment contract (88%) and worked full-time (57.6%). On average, participants worked 38.07 h per week (*SD* = 7.71; min. = 18; and max. = 68) and the mean organization tenure was 5.84 years (*SD* = 3.76; min. = 1; and max. = 17).

### 3.2. Measures

*Work engagement* was measured using the nine-item version of the Utrecht Work Engagement Scale (UWES-9) [49]. The UWES-9 includes three subscales, and each subscale consists of three items. Participants answered items such as: “When I get up in the morning, I feel like going to work” (*Vigor*), “My job inspires me” (*Dedication*), and “get carried away when I am working” (*Absorption*). All items were scored on a rating scale that ranged from 0 (*(almost) never*) to 6 (*(almost) always*). The internal consistency (Cronbach’s alpha) of the whole questionnaire was α = 0.92. Concerning the three subscales, in the current study the reliability coefficient was α = 0.83 for Vigor, α = 0.89 for Dedication, and α = 0.88 for Absorption.

*Workaholism* was assessed with the Dutch Work Addiction Scale (DUWAS) [50] that includes two five-item scales corresponding to the critical components of workaholism. Sample items are: “I find myself continuing to work after my co-workers have called it quits” (*Working Excessively*) and “I feel obliged to work hard, even when it’s not enjoyable” (*Working Compulsively*). Responses were given on a four-point frequency scale ranging from 1 (*(almost) never*) to 4 (*(almost) always*). The internal consistency reliability estimate using Cronbach’s alpha was α = 0.74 for Working Excessively, α = 0.71 for Working Compulsively, and α = 0.81 for the total score on workaholism.

*Obsessive–compulsive traits* were assessed using the items developed by Mudrak [51]. Three subscales were used: the Orderliness scale includes seven items (e.g., “I usually get through my work efficiently without wasting time”), the Rigidity scale consisted of six items (e.g., “I am usually consistent in my behavior, go about my work in the same way and frequent the same routes”), and the Superego reported five items (e.g., “I think that I have a more rigorous standard of right and wrong than most people”). All the items were rated on a seven-point agreement scale ranging from 1 (*strongly disagree*) to 7 (*strongly agree*). In the current study, Cronbach’s alpha was α = 0.82 for Orderliness, α = 0.77 for Rigidity, and α = 0.73 for Superego.

*Achievement orientation* was measured using the questionnaire developed by Lang and Fries [52] and included two scales. Hope of Success consists of five items (e.g., “I am appealed by situations allowing me to test my abilities”), and Fear of Failure includes five items (e.g., “If I do not understand a problem immediately I start feeling anxious”). Participants were asked to indicate their agreement with each item, using a four-point scale ranging from 1 (*strongly disagree*) to 4 (*strongly agree*). The internal consistency of the scales yielded a Cronbach’s alpha coefficient of α = 0.86 for Hope of Success, and α = 0.89 for Fear of Failure.

*Perfectionism* was evaluated using the Personal Standards subscale taken from the Frost Multidimensional Perfectionism Scale [32]. This scale includes seven items (e.g., “It is important to me that I be thoroughly competent in everything I do”). The response options varied on a five-point agreement scale ranging from 1 (*strongly disagree*) to 5 (*strongly agree*). The reliability of the scale was α = 0.71.

*Conscientiousness* was measured using the corresponding scale included in the short version [53] of the Big Five questionnaire proposed by Mowen [54]. This version of the conscientiousness scale consists of 3 characteristics (e.g., “I am an organized person”) rated on a 7-point frequency scale from 1 (*never*) to 7 (*always*). This scale reported an internal consistency coefficient of α = 0.88.

*Job demands* were assessed using the Job Content Questionnaire [44]. The scale includes nine items to assess demanding aspects of the job, such as time pressure and conflicting job demands. An example item is: “My job requires long periods of intense concentration”. Items were scored on a four-point Likert scale from 1 (*strongly disagree*) to 4 (*strongly agree*). In the current study, the reliability of the scale was α = 0.78.

*Overwork Climate* was measured with the Overwork Climate Scale (OWCS) developed by Mazzetti and colleagues [43]. Overwork *Endorsement* was assessed using the corresponding seven-item scale (e.g., “It is considered normal for employees to take work home”). The internal consistency reliability estimate using Cronbach’s alpha was α = 0.78. *Lacking Overwork Rewards* was measured with four items (e.g., “Overtime work is fairly compensated by extra time off work or by other perks”—*Reversed*). All items were rated on a five-point Likert scale ranging from 1 (*strongly disagree*) to 5 (*strongly agree*). Cronbach’s alpha for the scale was α = 0.71.

### 3.3. Strategy of Analysis

The research hypotheses were tested simultaneously with structural equation modeling (SEM) using the software package AMOS 21.0 [55]. Work engagement was included as a latent factor indicated by the observed levels of vigor, dedication, and absorption. In contrast, the critical dimensions of working excessively and working compulsively indicated the latent workaholism factor. The remaining variables were included as single indicators corresponding to the average score of the related scale, as the overall goal of the current research was to straighten out the difference among individual and contextual variables in explaining engagement and workaholism variance. Thus, the model included the measured personal characteristics (i.e., orderliness, rigidity, superego, hope for success and fear of failure), job demands, and the overwork climate components (i.e., overwork endorsement and lacking overwork rewards). Based on theoretical reasoning on the common ground shared by these variables, several correlations were allowed [56]. In particular, the model included correlations within personality factors and between job demands and overwork climate facets (i.e., overwork endorsement and lacking overwork rewards). In line with previous research [43], the current model allowed a cross-leading of the absorption dimension of engagement on the latent workaholism. This empirical evidence reflects the theoretical notion that workaholic and engaged employees are both intensely absorbed in their work, whereby time passes quickly, and are both reluctant to detach themselves from work [57]. To assess the model fit, the chi-square (χ2) statistic and the root mean square error of approximation (RMSEA) were used. We also examined fit indices less sensitive to sample size, including the Comparative Fit Index (CFI) and the Tucker Lewis Index (TLI). A model fit reporting values higher than 0.90 for TLI, CFI, and GFI, and an RMSEA equal to or lower than 0.08 is conventionally considered as acceptable [56].

## 4. Results

### 4.1. Descriptive Results

Table 1 presents descriptive statistics, internal consistencies, and correlations between the study variables. All the scales reported adequate internal reliability parameters, with values exceeding the minimum threshold of 0.70 [58]. In line with the theoretical framing as opposite forms of working hard, workaholism and work engagement were negatively related (*r* = −0.15, *p* = 0.001). All the significant relationships between the variables were in the expected direction, with a few exceptions. Among the components of obsessive–compulsive traits, rigidity was not significantly related to workaholism (*r* = −0.08, *p* = 0.060) and engagement (*r* = −0.01, *p* = 0.756). Furthermore, conscientiousness reported a significant relationship with work engagement (*r* = 0.27, *p* = 0.000), but not with workaholism (*r* = 0.02, *p* = 0.621).

### 4.2. Model Testing

**Figure 1:**
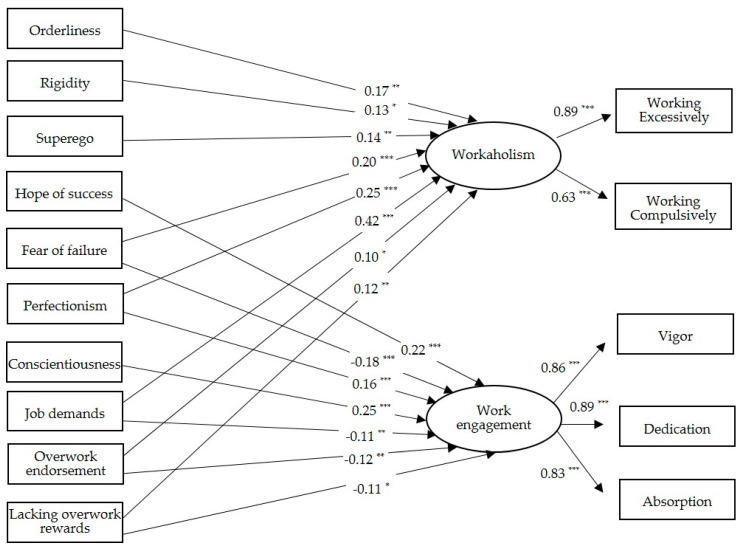
Structural equation results for the hypothesized model. **Note.** * *p* < 0.05; ** *p* < 0.01; *** *p* < 0.001.

The hypothesized model demonstrated a good fit to the data, *χ*^2^ (df = 55) = 158.42, *p* = 0.000; RMSEA = 0.06, SRMR = 0.04; GFI = 0.97; TLI = 0.92; CFI = 0.96. The final model is reported in Figure 1. For reasons of economy, correlations between errors of individual factors and organizational dimensions are not depicted. It should be noticed that the pathway from obsessive–compulsive traits and engagement was not significant. Orderliness reported a path coefficient of *β* = −0.09 (*p* = 0.111). In a similar vein, the path coefficient from rigidity to engagement was *β* = 0.01 (*p* = 0.831) and from superego to engagement was *β* = 0.06 (*p* = 0.184). On the other hand, these factors were positively associated with workaholism. To be specific, the paths coefficients on workaholism were equal to *β* = 0.17 (*p* = 0.004) for orderliness, *β* = 0.13 (*p* = 0.010) for rigidity and *β* = 0.14 (*p* = 0.005) for superego. Overall, these results provided partial support to *Hypothesis 1* and revealed that obsessive–compulsive traits are significantly and positively associated with a negative form of HWI, namely workaholism. On the other hand, these individual characteristics were not significantly associated with the positive type of heavy involvement toward one’s job, namely work engagement.

Among the achievement-oriented traits, hope of success did not report a significant association with workaholism (*β* = 0.01, *p* = 0.969), but it is also the factor characterized by the greatest association with work engagement (*β* = 0.44, *p* = 0.000). The component labeled as fear of failure reports an opposite relationship with the two forms of HWI. A higher fear of failure was associated to a greater workaholism (*β* = 0.20, *p* = 0.000), but also to low levels of engagement (*β* = −0.18, *p* = 0.000). Beyond the unexpected relationship between fear of failure and engagement, the current results supported the hypothesized association of workaholism with fear of failure. They indicated that higher engagement levels are related to hope of success, thus providing support to our second hypothesis (*Hypothesis 2*).

In the current sample, perfectionism was positively related to both workaholism (*β* = 0.25, *p* = 0.000) and work engagement (*β* = 0.16, *p* = 0.000). This evidence supported the assumption described in *Hypothesis 3* and concerning the link between perfectionism with opposite forms of HWI (i.e., workaholism and work engagement). Furthermore, conscientiousness reported a positive association with work engagement (*β* = 0.22, *p* = 0.000), but not significant with workaholism (*β* = −0.08, *p* = 0.137). These results partially confirm *Hypothesis 4* since it did not corroborate the assumption that a greater level of conscientiousness is related to higher workaholism scores.

As far as organizational factors are concerned, they all display significant but opposite association with workaholism and engagement. Workaholism was predominantly associated with job demands (*β* = 0.42, *p* = 0.000), but also with overwork endorsement (*β* = 0.10, *p* = 0.022) and lacking overwork rewards (*β* = 0.12, *p* = 0.005). Conversely, work engagement reported a negative relationship with job demands (*β* = −0.11, *p* = 0.013), overwork endorsement (*β* = −0.12, *p =* 0.007) and lacking overwork rewards (*β* = −0.11, *p* = 0.011). Overall, these findings provided support to our last hypotheses, namely *Hypothesis 5* and *Hypothesis 6*. The inspection of squared multiple correlations revealed that the tested model explained 40 percent of the variance in workaholism and 27 percent in work engagement.

## 5. Discussion

The current study aimed to delve deeper into the different weight of personality factors (i.e., obsessive–compulsive traits, achievement motivation, perfectionism, and conscientiousness) and situational factors (i.e., overwork climate and job demands) regarding two opposite forms of HWI, namely workaholism and work engagement. To this end, personality and organizational factors were assessed simultaneously, which means that the relationships of both categories with HWI are exclusive (i.e., controlled for the effect of the other set of variables). Although noteworthy theoretical models proposed in the workaholism literature identified this taxonomy concerning main antecedents of this compulsive drive to work [12,15,16], the concurrent investigation of these factors was substantially limited to the Big Five personality factors [2]. The obtained results pointed out different roles of individual and situational factors correlated to a heavy investment of time and effort in one’s work.

A first result suggests that obsessive–compulsive traits (i.e., orderliness, rigidity, and superego) were related to workaholism but not significantly associated with engagement. This evidence concerning the addiction to work corroborates previous results on obsessive–compulsive traits as drivers that increase the likelihood of developing addictions [40]. Obsessive–compulsive traits among workaholic employees translate into the inability to detach themselves from work-related activities and feelings of anxiety when they stop working. As for other addictions, compulsive working constitutes a coping or escape strategy against one’s obsession [59]. Workaholism shares intrusive thoughts (related to work), the inability to relax, and an intense concentration on oneself related to a failure in enacting effective coping strategies, such as seeking social support [60]. Our result could be explained by the workaholism conceptualization of Clark and colleagues [4], based on the following components: persistent thoughts around work, the expenditure of excessive energies in work-related activities, and harmful consequences on health and well-being. In the current study, these traits were not significantly related to work engagement. The current study provides additional support to earlier results, which attributed a prominent role to job resources, mainly located at the organizational level, in the etiology of engagement [61]. By excluding any relationship between obsessive–compulsive traits and work engagement, the current findings confirm the different motivational dynamics that prompt workaholic vs. engaged employees. Our results on the relationship between achievement motivation components and opposite forms of HWI draw a sharp distinction between the roots of workaholism and engagement. In the current study, fear of failure reported a positive association with workaholism. This result agrees with the seminal work proposed by Burke [62] on the role played by personal beliefs and fears in workaholism development. According to this author, compulsive tendencies constitute a core defining facet of workaholism entrenched in irrational or extreme beliefs. Among them, workaholic employees are incredibly concerned about perceiving themselves or being perceived as unsuccessful. Hence, they are driven to prove themselves by accomplishing admirable results. Thus, the current results corroborate the central role attributed by existing workaholism research to the intense fear of being judged as a failure [63]. Workaholic employees are not pushed to commit time to work out of intrinsic motivation, but rather to comply with the internal drive to employ any strategy to avoid failure. Fear of failure in meeting self- and others’ standards leads those employees to work harder, frequently check their work to prevent mistakes, continue thinking about work activities, and find potential improvement strategies [64].

On the other hand, the current study suggests a positive association between hope of success and work engagement. This evidence is consistent with previous results on autonomous regulation as a root mechanism leading engaged employees to invest extraordinary effort and time in work-related activities. Through a self-regulated process, engaged individuals freely choose to perform HWI because they enjoy their job and perceive it as enriching and stimulating [61]. This result agrees with the definition of work engagement as a harmonious passion, leading individuals to engage in a specific activity that is recognized as meaningful and represents them [65]. In contrast to workaholic employees, they do not experience an inner urge that compels them to center their existence on work-related activities as the only means for avoiding feelings of worthlessness. Overall, the positive association between fear of failure and workaholism, on the one hand, and hope of success and work engagement, on the other hand, concurs with workaholism corresponding to obsessive passion and engagement overlapping with harmonious passion [23]. Accordingly, obsessive passion (i.e., workaholism) arises when individuals are forced to undertake an activity by an internal contingency controlling their behavior. Conversely, harmonious passion (i.e., work engagement) refers to those individuals who freely pursue a significant goal with no contingency associated.

Concerning perfectionism, this study revealed a positive association with both workaholism and work engagement. On the one hand, the academic literature has widely proven the relationship between workaholism and perfectionism. These constructs share similar properties, which led several theoretical models to include perfectionism among the defining features of workaholism [66,67]. As previously described regarding the obsessive passion behind workaholism, these employees are also forced to overshadow highly urgent and critical tasks because they are overwhelmed by the obsessive pursuit of errors and detailed analysis of their work [68]. This conduct translates into poor job performance but, at the same time, strengthens their need to devote time to work. Therefore, our results align with previous findings suggesting the gap between the unrealistic expectations that perfectionists strive to meet and performance evaluations as a primary driver of workaholism [13]. In line with our hypothesis, work engagement also exhibited a positive association with perfectionism. This result corroborates prior consistent evidence of engagement association, especially with the facet of perfectionism defined as perfectionistic strivings [69]. Engaged employees are dedicated to fulfilling their performance standards due to the satisfaction derived from achieving relevant work goals. This strong commitment can relate to perfectionistic strivings, also known as personal standards perfectionism. In other words, the absorption dimension shared by workaholism and engagement could account for their positive relationship with employees’ levels of perfectionism.

Furthermore, only the positive form of HWI (i.e., work engagement) reported a significant relationship with conscientiousness. This evidence agrees with Schaufeli [2], who pointed out that engagement is positively related to conscientiousness but not significantly associated with workaholism. This result also agrees with studies assessing causal models of work engagement through longitudinal data and proving that conscientiousness can substantially enhance the level of engagement reported across different occupational sectors [41,70]. From a theoretical perspective, the characteristics of conscientious individuals could convincingly justify the relationship with engagement. Conscientiousness entails a strong sense of duty and self-discipline that also characterizes employees devoting a great effort to work-related activities to accomplish their goals [71]. Engaged employees are likely to report a strong conscientiousness, inspiring significant efforts to pursue their goals with responsibility and perseverance.

Along with assessing personality factors, the current study investigated the relationship of HWI with overwork climate and job demands (i.e., situational factors). To date, the research provided limited efforts to investigate the socio–cultural antecedents of workaholism. The comprehensive review of the existing literature performed by Sussman [12] found that situational factors could significantly contribute to workaholism development, such as being exposed to recurrent social modeling of compulsive work conduct or acting in organizational contexts with an excessive work ethic. Our results suggest that a significant amount of job demands report a remarkable association with workaholism. Prior empirical results pointed out this relationship and suggested that higher work pressure and problematic management practices require employees to spend greater effort and, consequently, contribute to workaholism development [60]. Our results on the association between demand overload and workaholism substantiate theoretical perspectives depicting this compulsive attitude toward work as the consequence of dysfunctional coping strategy, such as spending a greater amount of time and energy on work tasks [72]. The current results revealed a negative association between the perceived amount of demands and engagement. This evidence could be framed into the core assumptions of the Job Demands-Resources model [73]. Prolonged exposure to a critical amount of mentally demanding tasks with a high work pace drains employees’ energy progressively. Personal psychological resources consumption leads to a condition of fatigue that impairs their level of engagement [74].

Consistent with this result, the overwork climate components reported a negative association with engagement but were positively related to workaholism. Employees required to work long hours out of implicit organizational norms (i.e., overwork climate) are prone to display a compulsive conduct toward work. The combination of widespread overwork encouragement in the workplace, namely overwork endorsement, and the lack of organizational policies compensating the commitment of an extraordinary amount of time at work, defined as lacking overwork rewards, is conducive to workaholism and could jeopardize employees’ engagement. In line with previous studies, both negative and positive forms of HWI are significantly related to shared perceptions of expected conduct in the workplace [43]. Overall, these findings contribute to the conceptualization of workaholism as a form of overinvestment in work that, like work engagement, is highly related to the situational factors that characterize the work context.

### 5.1. Study Limitations and Recommendations for Future Research

This study makes significant contributions to research efforts that aim to obtain a detailed picture of the individual and organizational factors that act as potential antecedents of workaholism and work engagement. Nevertheless, this research has some limitations that must be considered. As a main limitation, the current study is based on cross-sectional data, which precludes establishing with reasonable certainty the causal link between personal characteristics, overwork climate, and job demands, on the one hand, and opposite forms of HWI, on the other. Conversely, this study has the merit of being a first attempt to concurrently assess the roles of different antecedents in explaining variance in workaholism and engagement. Future studies may enrich the current findings by gathering longitudinal data to draw unequivocal causal conclusions. A second limitation arises from the fact that our results were obtained through self-report measures. This choice is largely sustainable concerning the overwork climate since psychological climate entails an individual’s meaningful cognitive representation of work environment attributes, enabling them to decipher events in the organizational context and predict their consequences [75].

On the other hand, personality factors should be assessed through multiple sources to tackle the risk of common method bias affecting the relationship among the study variables. In line with Lievens [76], applying a multi-method approach to tackle the traditional overuse of self-report questionnaires could overcome the unspecific and context-free approach to measuring personal characteristics, leading to more informative results. In a similar vein, a future extension of the current model could embrace a multi-rater evaluation of workaholism and work engagement. Earlier results focusing on the multi-rater assessment of work engagement and workaholism have revealed a substantial agreement between employees’ and peers’ ratings on these forms of HWI [5].

Moreover, the obtained results suffer from relying on data collected within a single organization. Although participants offered a comprehensive overview of distinct roles in terms of responsibility, workload, and autonomy, it would be useful to replicate these findings in different organizational contexts to ensure their generalizability.

### 5.2. Practical Implications

The current study has several implications for stakeholders and practitioners willing to prevent workaholic behaviors and promote work engagement in organizational settings. According to our findings, strategies intended to modify situational factors should be prioritized to stimulate a work environment discouraging a compulsive drive to work (i.e., workaholism) but fostering employees’ healthy commitment (i.e., work engagement). Indeed, climate and job demands were conversely related to opposite forms of HWI, with demands reporting the strongest association with workaholism. Efforts to prevent workaholism and foster engagement by shaping situational factors and policies should be favored, considering the limited opportunities to affect employees’ characteristics. In line with previous results, an environment fostering the perception of overwork as a standard, rather than an exception rewarded with extraordinary compensation (i.e., overwork endorsement and lacking overwork rewards) should be a first concern to dissuade compulsive work-related behavior and enhance work engagement [43]. Individual conduct at work is strongly influenced by the perceived socio–cultural environment, particularly in terms of shared organizational norms related to expectations and rewarded behavior [77]. Moreover, recurrent patterns in managers’ and supervisors’ behavior serve as critical information sources on the normative order for proper conduct in a specific work context [78]. Accordingly, the implementation of leadership development programs could help shape values and practices that promote healthy commitment to one’s job and discourage the perception of excessive work patterns as a prerequisite for achieving productive output and accessing career advancement opportunities. The perception of managerial support emerged as a crucial resource that should be addressed to promote healthy work behavior and prevent undesirable conduct beyond the HWI inherent in workaholism, such as counterproductive work behavior and workplace bullying [79].

These measures could mitigate the behavioral manifestations of workaholism (i.e., working excessively) and inhibit widespread a climate that our results have shown to be inversely related to engagement. Limiting the requirement to overwork could also prevent a competitive environment where rewards result from comparing performance and evidence of involvement (e.g., time spent in the work setting) among colleagues [80]. Overwork climate should be replaced by a growth climate, where employees are provided with opportunities to evaluate one’s creativity to develop new personal and professional competencies [2]. A growth climate could inhibit the perception of an extraordinary number of job demands, which were most strongly associated with workaholism in the current study. An environment characterized by opportunities to acquire skills and undergo training also supports employees’ capacity to tackle job demands without feeling compelled to make their tasks more challenging to prove their abilities [81]. As a further implication, employees’ psychological well-being could be fostered through novel approaches targeting behavioral/brain interventions, such as neurodidactics [82]. Additionally, job redesign approaches should include managers’ and supervisors’ constant monitoring of the demands faced by employees [83]. Maintaining a proper amount of job demands can function as a protective measure against compulsive work patterns and ensure the presence of engaged employees.

## 6. Conclusions

Noteworthy literature reviews and meta-analyses on workaholism provided comprehensive descriptions of individual characteristics and situational factors behind this compulsive inner drive to work. This research intended to enrich the existing literature by concurrently analyzing several individual and situational factors recognized as significant in the etiology of workaholism, also including a positive form of HWI (i.e., work engagement). Despite the limitations of a cross-sectional design in establishing causal relationships, this research constitutes one of the first attempts to identify which correlates would explain the most variance in workaholism and work engagement between personality factors, organizational climate, and job demands. The strong and reverse associations of organizational climate and job demands with opposite forms of HWI suggest valuable inputs for progress in workaholism prevention and engagement enhancement strategies.

## Figures and Tables

**Table 1:** Means (*M*), standard deviations (*SD*), internal consistency estimate (in parentheses), and correlations among the study variables (*n* = 523).

Variable			*R*											
	*M*	*SD*	1	2	3	4	5	6	7	8	9	10	11	12
1. Workaholism	2.43	0.53	(0.81)											
2. Work Engagement	5.62	0.90	−0.15 **	(0.92)										
3. Orderliness	5.58	0.97	0.13 **	0.19 ***	(0.82)									
4. Rigidity	4.56	1.10	0.08	−0.01	0.37 ***	(0.77)								
5. Superego	5.62	0.88	0.22 ***	0.17 ***	0.47 ***	0.48 ***	(0.73)							
6. Hope for Success	3.63	0.45	0.07	0.31 ***	0.25 ***	−0.02	0.25 ***	(0.86)						
7. Fear of Failure	2.41	0.78	0.19 ***	−0.21 ***	−0.07	0.29 ***	0.03	−0.14 **	(0.89)					
8. Perfectionism	3.41	0.59	0.32 ***	0.20 ***	0.09 *	−0.05	0.16 ***	0.22 ***	−0.08	(0.71)				
9. Conscientiousness	5.56	1.13	0.02	0.27 ***	0.68 ***	0.27 ***	0.34 ***	0.16 ***	−0.04	0.03	(0.88)			
10. Job Demands	2.70	0.48	0.39 ***	−0.13 **	−0.07	0.04	0.10 *	−0.06	0.08	0.15 **	−0.07	(0.78)		
11. Overwork Endorsement	2.20	0.81	0.25 ***	−0.16 ***	−0.03	0.09 *	0.07	−0.03	0.02	0.16 ***	−0.09 *	0.31 ***	(0.79)	
12. Lacking overwork rewards	3.47	0.98	0.19 ***	−0.14 **	0.04	0.09 *	0.02	0.02	0.02	0.04	0.32 ***	0.13 **	0.33 ***	(0.71)

Note. * *p* < 0.05; ** *p* < 0.01; *** *p* < 0.001.
